# Regulation of genomic and biobanking research in Africa: a content analysis of ethics guidelines, policies and procedures from 22 African countries

**DOI:** 10.1186/s12910-016-0165-6

**Published:** 2017-02-02

**Authors:** Jantina de Vries, Syntia Nchangwi Munung, Alice Matimba, Sheryl McCurdy, Odile Ouwe Missi Oukem-Boyer, Ciara Staunton, Aminu Yakubu, Paulina Tindana, the H3Africa Consortium

**Affiliations:** 1 Department of Medicine, Faculty of Health Sciences, University of Cape Town, Cape Town, South Africa; 2 Department of Clinical Pharmacology, College of Health Sciences, University of Zimbabwe, Harare, Zimbabwe; 3 Center for Health Promotion and Prevention Research, School of Public Health, University of Texas Health Science Center at Houston, Houston, USA; 4 Cameroon Bioethics Initiative (CAMBIN), Yaounde, Cameroon; 5 University of Stellenbosch, Stellenbosch, South Africa; 6 National Health Research Ethics Committee, Federal Ministry of Health, Abuja, Nigeria; 7 Navrongo Health Research Centre, Ghana Health Service, Navrongo, Ghana

## Abstract

**Background:**

The introduction of genomics and biobanking methodologies to the African research context has also introduced novel ways of doing science, based on values of sharing and reuse of data and samples. This shift raises ethical challenges that need to be considered when research is reviewed by ethics committees, relating for instance to broad consent, the feedback of individual genetic findings, and regulation of secondary sample access and use. Yet existing ethics guidelines and regulations in Africa do not successfully regulate research based on sharing, causing confusion about what is allowed, where and when.

**Methods:**

In order to understand better the ethics regulatory landscape around genomic research and biobanking, we conducted a comprehensive analysis of existing ethics guidelines, policies and other similar sources. We sourced 30 ethics regulatory documents from 22 African countries. We used software that assists with qualitative data analysis to conduct a thematic analysis of these documents.

**Results:**

Surprisingly considering how contentious broad consent is in Africa, we found that most countries allow the use of this consent model, with its use banned in only three of the countries we investigated. In a likely response to fears about exploitation, the export of samples outside of the continent is strictly regulated, sometimes in conjunction with regulations around international collaboration. We also found that whilst an essential and critical component of ensuring ethical best practice in genomics research relates to the governance framework that accompanies sample and data sharing, this was most sparingly covered in the guidelines.

**Conclusions:**

There is a need for ethics guidelines in African countries to be adapted to the changing science policy landscape, which increasingly supports principles of openness, storage, sharing and secondary use. Current guidelines are not pertinent to the ethical challenges that such a new orientation raises, and therefore fail to provide accurate guidance to ethics committees and researchers.

## Background

The introduction of genomics and biobanking methodologies to the African research context through platforms such as MalariaGEN [1], 1000Genomes [2] and H3Africa [3] has simultaneously introduced some of the ethical challenges associated with it [4, 5]. Underlying such research is a shift in the way in which research is conducted, towards greater openness, sharing of resources, collaboration between scientists from across the world, and re-use of samples and data for secondary research. This shift has introduced a requirement to reconsider some of the key ethical principles and practices of health research. Most notably, narrow conceptualizations of informed consent as constituting an agreement to participate in one research project with a clearly defined question are not tenable in such research and are gradually being substituted with an acceptance that broad consent—or at least tiered consent—areethically acceptable and more appropriate consent models to be used in such research [6, 7]. Related to this shift is greater focus on considering the governance of scientific resources, for instance through data sharing policies that promote ethical best practice [8].

Yet these changes are often not incorporated in the regulatory documents that guide researchers and ethics committees. In assisting H3Africa researchers [3] navigate the landscape of ethical regulations, guidelines and ethics review across the continent, it became clear that African regulation is either absent, outdated, conservative or difficult to navigate. Simultaneously, the ethics committees that apply these regulations tend to be very cautious about approving genomics and biobanking study proposals, particularly where these involve proposals to use a broad consent model or to share samples for unspecified future use with researchers from across the globe. Such a wary approach is not surprising given the history of biomedical interventions across the continent [9–11], which has led to a mistrust of international collaborations and a perceived need to protect local scientists and populations from outside research agendas. Most pertinently, such past experiences offered little opportunity for African scientists to intellectually engage in, or lead, African health research, and often reduced their contribution to operational tasks such as for instance those relating to sample collection [12]. These past practices inform current legislation, policies and tentative responses to unknowns involved in genomic research [13]. In response to these challenges, we set out to not only work with members of ethics committees and National Ethics Councils to explore ethical challenges around genomic research and biobanking [14, 15], but we also put together a comprehensive analysis of the existing ethics regulatory framework for genomics and biobanking research, which we report in this paper.

In our review, we built on other similar although more restricted reviews published in the literature. A 2013 study [16] reviewed international and African guidelines, policies and standard procedures for genomic research across 11 African countries to document how they address issues of confidentiality, import/export of samples, secondary use of samples and informed consent. In 2012, Sather and Dhai documented how guidelines in 6 African countries (5 of which overlapped with Staunton and Moodley [16]) compare to those of higher income countries and countries in the BRICS zone [17]. Arguably the most comprehensive analysis of national regulatory guidelines was performed by [18] who documented how regulation in three African countries (also covered in the other sources) addressed consent to and authorization for export of human biological material collected as part of research. All three studies showed that the guidelines differed considerably and Staunton and Moodley concluded that this could negatively affect collaboration in genomic research. Santhar and Dhai reported that most African countries as well as countries in the BRICS region preferred specific consent unlike other developed countries. These three studies collectively reviewed documents from 12 African countries. Recent publications detailed the regulation of genomics and biobanks in Zambia [19], South Africa [20] and Nigeria [21], which we included in our analysis.

In this paper, we report the results of a review that had the aim to document whether and how African country guidelines and policy documents discuss genomic research and biobanking research, and we specifically explored provisions for the use of broad consent to support such research. The analysis of the review focused on permitted informed consent types, guidance on the reuse of samples collected as part of research, sample storage, ownership of samples collected as part of research and the export of samples collected as part of research.

## Method

We sourced ethics documentation from the countries involved in H3Africa research through personal contacts, bioethics databases and Google. We first searched all the online ethics documents databases or repositories known to us. We searched the UNESCO Global Ethics Observatory (GEObs) [22], the Health Research Web (HRWeb) [23], the 2015 edition of the International Compilation of Human Subjects Standards compiled by the US Office of Human Research Protections, [24] the Training And Resources In Research Ethics Evaluation (TRREE) database, [25] and the ClinRegs database [26]. To cast our net as wide as possible, we also googled ethics guidelines and regulations on human subjects’ protections, genetic/genomic research and biobanking research in Africa. Where we felt there was a gap in our information, we googled the country name together with selected keywords, for instance ‘Namibia, export, biological samples’. In this way, we identified a few additional sources including a recent Kenyan ethics guideline dealing with sample export and storage. Whilst there is an important difference between laws and other regulatory ethics documents (in the sense that the first are mandatory, the second may not be), we were interested primarily in ethical guidance for health research. For this reason, in our search we primarily focused on the identification of regulatory ethics documents (guidelines, SOPs and so forth). In countries that did not have ethics guidelines but that had laws prescribing good ethical practice—such as is the case in Zambia, Senegal and Benin for instance—we included such laws in our study.

Finally, we contacted H3Africa researchers and members of research ethics in 21 African countries and requested copies of any guidelines we did not yet have. Specifically, we targeted researchers and members of ethics committees attending the H3Africa Ethics Consultation meetings [14], at which ethics committees from over 20 African countries were present. During the second H3Africa Consultation Meeting, which was held in Zambia in May 2015 [15], we circulated lists of all the documents we had sourced for each country and asked meeting attendants to check for completeness. In this way, we sourced an additional few documents that we had not obtained through our other search strategies, including national ethical guidelines from Ghana which are still in their draft form.

All guidelines were imported into NVivo 10 [27] for thematic analysis using a pre-defined coding scheme. The coding scheme covered themes on consent, ownership of samples, destruction of samples, international guidelines, sample and data sharing. The coding scheme was discussed by members of the H3Africa working group on ethics and documents in NVivo were coded by two researchers (JDV, NSM). Because not all researchers had access to NVivo, we exported the coding scheme into Excel and asked the contributing authors to summarize pertinent aspects of the guidelines they were analysing in the Excel spreadsheet, and to summarize the guidelines’ position on issues pertinent to genomics and biobanking research. Where we were unsure about the interpretation of the guidelines, we sought advice from our contacts in the countries concerned. This was particularly pertinent in the case of Ethiopia, where there is ongoing ambiguity about the applicability of biodiversity legislation on human genetic research.

## Results

We collected a total of 30 documents from 22 African countries (see Table 1 for an overview). The type of documents—and thus the sources of ethics guidance—varied tremendously across countries. They included: standard operating procedures; national guidelines for health research; national guidelines for genetic research; ministerial decrees and laws. Most of the documents were published in the last 5 years. The oldest was published in 1997 in Guinée, while the latest was published in 2015 in South Africa. The Ethiopian guidelines were revised in 2014 primarily to include aspects of genetic and collaborative research—we only reviewed the second edition of those guidelines. The documents we reviewed were published by different authorities, including Ministries of Health, National Ethics Committees, National Councils for Science and Technology, Parliament and an Office of the President. Where countries had ethics regulations or guidelines interpreting the legal framework, we only examined those without also examining the laws impacting on health research. In the following, we detail those instances in which country regulations *do* address particular topics. If countries are not mentioned under the different topics discussed in the remainder of the paper, then this is because they are silent on those topics.

**Table 1:** List of countries and documents included in the analysis

Country	Nature document	Title of document	Year of Publication
Benin	Law	Code D’ Éthique et de Déontologie pour La Recherche en Santé en République du Bénin.	2010
Botswana	National guidelines	Standard Operating Procedures for Review Of Biomedical and Bio-Behavioural Research in Botswana	2011
Cameroon	SOP	Standard Operating Procedures for Research Ethics Committees (RECs) in Cameroon	2012
Ethiopia	National guidelines	National Research Ethics Review Guideline Fifth Edition	2014
Ghana	National Guidelines	Draft National Ethics Guidelines and Standard Operating procedures of Institutional Review Boards	In Development
Guinea	SOP	Procédure Opératoire Standardisée d’Examen d’un Projet de Recherche Biomédicale	2013
		Livre Troisieme de l’Éthique pour la Recherche en Santé	1997
Kenya	National guidelines	Guidelines for Ethical Conduct of Biomedical Research Involving Human Subjects in Kenya	2004
Lesotho	SOP	Standard Operating Procedures for the National Health Research Ethics Committee	2013
Malawi	National Guidelines for genetic research	Policy Requirements, Procedures and Guidelines for the Conduct and Review of Human Genetic Research in Malawi	2012
Mauritius	Law	The Clinical Trials Bill	2010
	National guidelines	Ethical Guidelines for Biomedical Research Involving Human Subjects	2003
Namibia	National guidelines	Guidelines on Clinical Trials in human subjects	2003
		Research Management Policy	2003
Nigeria	National guidelines	National Code of Health Research Ethics	2007
		Policy Statement on Storage of Human Samples in Biobanks and Biorepositories in Nigeria	2013
Rwanda	SOP	National ethics Committee Standard Operating Procedures	2009
Senegal	Law	Code d’Éthique pour la Recherche en Santé	2009
	Ministerial decree	Arrêté Portant Adoption des Bonnes Pratiques Cliniques (BPC ICR) pour la Conduite des Essais Cliniques.	2011
		Arrêté Ministeriel Portant Adoption du Guide du Chercheur et de la Brochures des Membres du CNERS pour l’Évaluation et le Suivi des Protocols de Recherche	2013
Sierra Leone	National guidelines	Guidelines for conducting clinical trials of medicines, food supplements, vaccines, and medical devices in sierra Leone.	Not Stated
		Guideline For Good Clinical Practice (GCP) In Sierra Leone	
South Africa	Law	National Health Act	2003
	National guidelines	Department of Health Guidelines, 2nd edition	2015
		MRC guidelines on Ethics for medical research, reproductive biology and genetic research	2002
Sudan	National guidelines	National Guidelines for Ethical Conduct of Research Involving Human Subjects	2008
Tanzania	National Ethics Guidelines	Guidelines of Ethics for Health Research in Tanzania	2009
Togo	Ministerial Decree	Charte du Comité de Bioéthique pour la Recherche en Santé	2009
Uganda	National Guidelines	National Guidelines for Research Involving Humans as Research Participants	2014
Zambia	Law	The National Health Research Act	2013
Zimbabwe	National guidelines	Ethics Guidelines for Health Research Involving Human Participants in Zimbabwe	2011

*SOP* Standard Operating Procedure

### Nature of documents

Of the countries we included in our analysis, only (Malawi, Nigeria and South Africa) had specific national or local guidelines for genomic and/or biobanking research. Malawi and Nigeria both had national guidelines published as addendums to the National Health Research Ethics Guidelines. The Malawian addendum focuses on human genetic research, whilst the Nigerian addendum focuses on storage of human samples in biobanks. In South Africa one of the university ethics committees had developed a local guideline for biobanking research, which we included in our analysis.

Of the countries we included in our analysis, only Benin and Senegal seem to have regulated health research through specific Health Research Ethics laws. Zambia has a Health Research Act which also contains large sections relating to research ethics, whilst in most other countries health research and research ethics are regulated through different laws for instance those pertaining to health and healthcare. In a number of countries, for instance in South Africa and Nigeria, the Health Acts devolve responsibility for development of ethics guidelines to national research ethics councils. Only the Zambian Health Research Act published in 2013 specifically mentions and regulates genomics and biobanking research. In the other countries, insofar as we are able to assess, genomics and biobanking are not specifically mentioned or addressed in the legal framework.

In all the other countries, provisions for genomics and biobanking were incorporated in the main national ethics guidelines, either deserving separate description or exceptional status (such as in Rwanda, Ethiopia and draft guidelines for Ghana), or by implication (such as Cameroon) because the guidelines discuss topics pertinent to genomics and biobanking, such as sample sharing or broad consent.

### Consent for genomic research

There appears to be increasing international acceptance for broad consent to be the ‘best compromise’ consent model to promote the sharing of scientific resources, balancing patient preferences, participant protection, and the utility of data and samples that are collected [28]. Specifically for genomics and biobanking research, some work has gone into exploring whether and when the use of broad consent is ethical, both internationally [6] and in Africa specifically [7]. Being the pillar of ethics regulation of health research worldwide, it is hardly surprising that informed consent was discussed in all the documents we reviewed. There is, however, tremendous difference in the way in which informed consent is described—from being more abstract and aspirational as in the case of the guidelines from Kenya, to being very detailed and descriptive as in the case of guidelines from Malawi.

The use of broad consent for future unspecified uses is specifically prohibited only in Zambia, where the 2013 Health Research Act stipulates that “A person shall not withdraw blood, blood products, tissue or gametes from a living person for any unspecified future health research activity or unspecified storage” (Zambia Health Research Act, provision 47(2))—a provision that is currently applied by Zambia’s National Health Research Ethics Council (James Munthali, personal communication). The potential effect of this provision to effectively block health research in the country led some Zambian authors to call for a revision of the Health Act [19]. If samples need to be stored for research that is in line with the original research for which samples were collected, then Zambian researchers are required to use a separate consent form. Although the use of broad consent is not specifically discussed or prohibited in Malawi and Tanzania, the guidelines for these two countries also suggest that it may not be used. In Malawi, the collection of samples for unspecified future use is not allowed—which suggests that the use of broad consent would also not be permissible. In Tanzania, the 2009 Guidelines for Health Research forward the position that “consent is provided for the intended study, and it is unethical to use specimens for purposes other than those consented for” (pg 86, section 8.8). Where researchers want to use stored samples for secondary research, the Tanzanian guidelines require that researchers submit a new protocol for ethics review. Although the document does not describe whether participants ought to be recontacted for consent, but that seems to be the intent of the 2010 Material Transfer Agreement (MTA). The 2010 Tanzanian MTA form notes that those involved “shall use all reasonable efforts to maintain the consents in effect once obtained” (pg 6, article VI.a).

In the majority of countries (Benin, Ghana, Guinee, Kenya, Lesotho, Mauritius, Namibia, Swaziland, Togo and Zimbabwe), the description of consent requirements is generic, and broad consent is neither prevented nor promoted. The absence of specific stipulations in the guidelines for these countries suggests that broad consent could possibly be used. In Kenya, the national guidelines stipulate that if the identity of the sample donor is known, then they should be re-consented for future use or the ethics committee must provide a waiver of this requirement. This latter requirement was strengthened in more recent (2014) guidelines for the export and storage of human biological samples, in which it is stipulated that sample donors should preferably consent to sample export (and, presumably, storage) but if for some reason they have not, then researchers should seek a waiver from the ethics committee.

In the guidelines from the remainder of the countries (Botswana, Sierra Leone, Senegal, Uganda, Cameroon, Nigeria, South Africa, Sudan, Rwanda, Nigeria and Ethiopia), consent for future unspecified research is mentioned and allowed, but with conditions attached. Guidelines from Botswana, Sierra Leone, Senegal and Uganda require that a separate form be used to seek consent for any future use—which looks like a regulatory preference for tiered consent. Guidelines for Cameroon, Nigeria, South Africa and Sudan do not require separate consent forms for future use, but rather stipulate that participants need to be informed of intentions to store samples for future research. A caveat in Sudan is that the regulations require that participants are re-consented at ‘regular intervals’, without specifying what a reasonable timeline for re-consenting would be. This provision is not specific to genomic research and biobanking however; participants in ongoing epidemiological research also need to be re-consented at regular intervals, even when the study aims remain the same. Finally, the guidelines for Rwanda, Nigeria and Ethiopia specify that broad consent can be used for the collection of samples for future use, but leave it up to ethics committees to decide on the appropriateness of the consent models proposed in research. Nigeria is the only country that specifically mentions and allows ‘broad consent’, which is defined as “consent in which the type or purpose of research is defined in broad terms and for a work that is not specified by time” (Section E.2 of the Policy Statement). On the contrary, blanket consent, which is defined as consent “in which the type or purpose of the research is not defined in any way and does not restrict the use of donated specimen to any type of research” (ibid) is not allowed.

### Storage of samples

Whilst sample storage is allowed in all countries (explicitly or implicitly), only few countries offer specific guidance on the timeframe for storage. In Zambia, samples can only be stored for a period not exceeding 10 years and permission is required for storage longer than 10 years. Samples can only be stored in designated research facilities. In Malawi, samples cannot be stored for more than 5 years. Research specifically aimed at storing human biological materials for future research or retrospective genetic analysis is not allowed in Malawi. Guidelines from Zimbabwe describe that extraterritorial storage of samples beyond the study period is not allowed. It is not clear how the national regulator ensures compliance with this provision.

### Re-use of samples

In the documents we reviewed, those from 14 countries specifically address issues of re-use of samples collected as part of research. In Botswana, Ghana, Ethiopia, Rwanda, Uganda, Kenya, Nigeria, Senegal, Sudan and Tanzania re-use of samples requires approval from an ethics committee. The other countries are silent on whether ethics approval is required for re-use. In Botswana, the formal requirement is for sample donors to be re-consented before their samples are used again; however this requirement can be waived by an ethics committee. In Ethiopia, the sharing of samples without consent, ethics approval and an MTA is mentioned as a specific example of research misconduct. In Rwanda the re-use of samples is subject to approval by the National Health Research Ethics Committee. In Malawi, sample collection for future use is not allowed, which suggests that re-use of samples is also not allowed. In Uganda, decisions on the re-use of samples collected as part of research will be determined by the institution that has custodianship of the samples. Although provisions for the re-use of samples are mentioned in the Zambian Health Research Act 2013, these do not describe who can access stored samples, for what purposes, and whether secondary use requires ethics approval.

### Export of samples and international collaboration

In contrast to provisions around sample storage and re-use, the export of samples is rather tightly controlled in many African countries. The guidelines from ten countries offer explicit guidance for the export of samples, and in all of these countries researchers require approval or permission from one or more national agencies, for instance from a national ethics review body (Ethiopia, Lesotho, Nigeria and Rwanda); from a national regulatory authority (Botswana, Malawi, South Africa and Zambia); from a Ministry of Health (Cameroon and Zambia); or from a national body for health research, medical research or for science and technology more broadly (Kenya, Uganda and Zimbabwe).

National-level approval is usually required in addition to (or as part of) ethics approval for the primary collection of the samples. In some cases, this national approval or permission takes the form of export permits only (such as is the case in South Africa, see [20]). In other countries (such as Ethiopia and Zimbabwe), researchers need to obtain both national-level ethics approval and an export permit.

Some countries (Ethiopia, Malawi, Kenya and Zambia) require that a local PI be associated with any research on country samples or data taking place outside the country while in Nigeria the PI must be affiliated to a registered institution in Nigeria capable of doing the proposed study. Malawi and Zambia require that all foreign-based researchers be affiliated to one of the local research institutions while in Zimbabwe, foreign researchers are required to obtain registration from the Research Council of Zimbabwe.

Nine countries (Botswana, Cameroon, Ethiopia, Ghana, Kenya, Rwanda, Uganda, Zambia and Zimbabwe) specifically mention and endorse international collaboration—guidelines for the other countries are silent on this topic. Guidelines for Botswana, Kenya and Uganda stipulate that export of samples is only allowed when there is no capacity in the country to conduct the same analyses—a requirement that appears to be quite strictly enforced in at least Botswana. These provisions are accompanied by a strong recommendation (Uganda) or requirement (Botswana and Kenya) that local capacity is built or strengthened where the export of samples is concerned. These nine countries require that international collaborative research be responsive to the health needs of the population. In Botswana, Ethiopia and Kenya, international collaboration needs to lead to capacity building. Malawi and Lesotho do not offer guidance for international collaboration whilst they do offer guidance for the export of samples—which implies international collaboration. In Ethiopia and Kenya, a local co-investigator needs to be included in all future studies making use of stored or exported samples. In Tanzania, if there is local technology for analysis in country, the researcher must explain why the samples are being sent out of the country. They must note whether or not a local Tanzanian is involved in analysis. In Malawi, export of samples is discouraged.

### Data and sample sharing

Only guidelines from Cameroon, Ethiopia and Tanzania specifically mention data sharing. Cameroon and Tanzania require a Data Sharing Agreement to be submitted as part of the ethics review application. Ethiopian guidelines stipulate that an Ethiopian ethics committee needs to review all secondary studies, also if samples are stored abroad.

### Ownership of samples

Ethics documents from seven countries refer to ownership of samples collected as part of research. In Zambia, the Minister of Health, in consultation with the National Health Research Authority, is responsible for making a decision on ownership of biological materials, including derivatives and modifications, collected as part of research. In Malawi, samples remain the property of the Ministry of Health. In Botswana, Ghana, Ethiopia, Rwanda and Uganda the institution that collected the samples has custodianship of the samples and holds the samples in trust on behalf of the research participants and the government.

Cameroon and Nigeria are not prescriptive about sample ownership, but guidance indicates that decisions on ownership of samples have to be agreed by local investigators and their collaborators before the start of the research and a copy of the agreement needs to be submitted to the ethics review committee.

### Return of genetic results

Documents from seven countries (Botswana, Cameroon, Ethiopia, Rwanda, Malawi, Sudan and Uganda) specifically refer to return of genetic results. One important concern across these countries seems to be the impact of genetic research on family members, and whether or not these should also be included in feedback. For instance, Ethiopian guidelines stipulate that individual research results should not be given to family members or third parties without written permission from the subject and approval by the National Health Research Ethics Council. In Malawi, return of genetic results would be determined by the investigator based on the sensitivity and specificity of the test and participants would have to consent to disclosure of results. If genetic study results are shared then this has to be done by a genetic counsellor. In Botswana and Cameroon, when return of results is planned, participants should be asked to decide whether or not they want to receive individual genetic test results. In Cameroon, the feedback of genetic study results will also require genetic counselling and participants must be informed whether the test is available outside the research context and who would have access to the study results. In Rwanda results of genetic test are not to be divulged to third parties unless properly authorized. However, the authority required for giving permission is not indicated. In Uganda, any result that are of clinical relevance, including incidental findings, must be fed back to study participants. In Sudan, participants need to be informed, during the consent process, of the policy in place for feeding back genetic test results and the precautions in place to prevent unauthorized disclosure of a participants’ genetic study results.

## Discussion

In this review, we examined the existing ethics regulatory framework for genomic research and biobanking in 22 African countries. Guidance for genomic research and biobanking takes place at three levels: considerations of genomics and biobanking are either completely absent from the regulatory environment, incorporated within the broader ethics framework for health research, or published as separate texts. The absence of regulation for genomic research in Africa is not peculiar to genomic research but biomedical research as a whole, [29] note that most countries are still developing standard operating procedures for their research ethics committees and some countries do not have existing guidelines for health research despite ongoing research in these countries. This may explain why we were only effective in sourcing guidance documents from 22 countries out of the 54 that make up the continent—there may not have been any in the other countries.

The study shows that just three countries have specific guidelines for genomic or biobanking research. Overall, many of documents we analysed seem to have been developed in response to clinical trials, epidemiological research or more broadly to health research—and the principles put forth are then taken to apply to genomic research and biobanking in the same way. This extension is problematic because these ethical principles do not accommodate changing scientific practices which involve the sharing and secondary use of resources globally, including associated changes in consent.

Where specific ethics guidelines are absent, researchers and ethics committees are left to make their own decisions, without a framework for their deliberations and decisions. The risk is that fears and misunderstanding could then guide decision-making—which would hardly be conducive to thorough ethical reflection. We found that in those countries where guidelines are very specific, they run the risk of becoming quickly outdated in the context of rapidly evolving research. This is the case for Malawi and Zambia, for instance, where very specific provisions for genomic research and biobanking appear to have started to obstruct genomic research in those countries.

Discussions with ethics committees in the context of H3Africa research (de Vries et al, [14, 15, 30] have suggested that many ethics committee members struggle with the notion of ‘broad consent’—the type of consent required to allow broad re-use of samples and data for secondary research. It is therefore surprising that our analysis revealed that broad consent is (apparently) allowed in all but three countries (Zambia, Malawi and Tanzania). In Zambia, the use of broad consent is illegal according to the Health Act 2013; in Malawi and Tanzania, the language used in the ethics documentation suggests that broad consent is not allowed. In all the other countries we examined, guidelines are either silent on the type of consent that can be used for genomic research, they allow consent for ‘future unspecified use’, or specifically endorse broad consent as a valid consent model in health research. This trend is encouraging as it means that there is no a priori reason to be concerned about the possibility that broad consent can be used in the majority of African countries [7].

One defining characteristic of genomics and biobanking studies is the sharing of resources, including samples and data, for secondary use globally. An essential and critical component of ensuring that secondary use is aligned with ethical principles and best practice is in regulating secondary use through governance. It is therefore problematic that data and sample sharing are most sparingly covered in the guidelines with only Cameroon, Ethiopia and Tanzania incorporating sections that focus on data sand sample sharing. Whilst ten countries have specific guidance for export of samples, and nine countries specifically mention and allow international collaboration, beyond MTAs none of the countries require a description of the governance of secondary research including sample and data sharing policies.

Guidelines for only three countries were specific to genetic, genomic or biobanking research. Although guidelines in many other countries describe regulations for export of samples for instance, we suspect that in most cases these regulations pertain to the sharing of samples between collaborators, for specific projects. The sharing of samples in the context of biobanking research—including the export of samples for biobanking elsewhere, or the export of cell lines—may not have been intended or imagined when guidelines were drafted. This complicates the applicability of existing guidelines to such research. Of the countries that specifically mention export of samples, many require national-level approval in addition to ‘local’ ethics review; a justification for why the work cannot happen in-country; a document regulating the exchange (export permit, MTA) and sometimes involvement of a national researcher in the overseas work. Interestingly, the guidelines are completely silent on ethical issues or responsibilities associated with the import of samples into African countries.

A limitation of this paper is that it focuses solely on ethics regulatory documents and not on ethics review practices. These are obviously different and the difference is important: for instance, although the Tanzanian and Malawian documents we perused suggest that broad consent is not allowed, we know that ethics committees in these countries have in the past approved studies that used a broad consent model. Similarly, although the use of broad consent is apparently allowed in most African countries, within H3Africa there have been several instances of ethics review committees questioning whether broad consent is ethical. It is important therefore to go beyond document analysis to investigate actual review practices and what ethics committee members believe is written in the regulatory documents. In a similar vein, our focus on mostly national documents, with few institutional or committee policies (Ghana, South Africa and Tanzania), also means that we might have missed important nuances in the way in which national policies are implemented or interpreted locally. An example would be the absence of a national requirement for Material Transfer Agreements in South Africa—most research institutions in this country would require these in any case, even if the national regulator does not.

## Conclusion

Overall, in the rapidly changing landscape of science—epitomised in the fields of genomic research and biobanking—ethics guidelines need to be broad and flexible enough to accommodate changes, whilst also offering guidance on the principles that should be applied to foster ethically sound health research. Key principles that ought to be incorporated into African guidance for genomic research and biobanking relate to promoting African leadership and ownership of genomics and biobanking science and capacity strengthening as an essential feature of international collaboration. In terms of specific guidance supporting ethics committee decision-making, we think that what is required are guidelines that address issues relating to sample and data sharing and the requirements of governance frameworks supporting these. What is also required is a clear statement, by African governments, national health ethics councils or other authorities charged with developing the ethical frameworks for research, about the appropriateness of using broad consent in the context of African genomics and biobanking research.

## Abbreviations

GEObs: UNESCO Global Ethics Observatory
HRWeb: Health Research Web
MTA: Material transfer agreement
TRREE: Training and Resources in Research Ethics Evaluation
